# Molecular Origins of the Mendelian Rare Diseases Reviewed by Orpha.net: A Structural Bioinformatics Investigation

**DOI:** 10.3390/ijms25136953

**Published:** 2024-06-25

**Authors:** Anna Visibelli, Rebecca Finetti, Neri Niccolai, Ottavia Spiga, Annalisa Santucci

**Affiliations:** 1 Department of Biotechnology, Chemistry and Pharmacy, University of Siena, 53100 Siena, Italy; anna.visibelli2@unisi.it (A.V.); rebecca.finetti@student.unisi.it (R.F.); ottavia.spiga@unisi.it (O.S.); annalisa.santucci@unisi.it (A.S.); 2 Le Ricerche del BarLume Free Association, Ville di Corsano, 53014 Monteroni d’Arbia, Italy; 3 Industry 4.0 Competence Center ARTES 4.0, Viale Rinaldo Piaggio, 56025 Pontedera, Italy

**Keywords:** rare diseases, missense pathogenic variants, protein structure, databank analysis, structural bioinformatics

## Abstract

The study of rare diseases is important not only for the individuals affected but also for the advancement of medical knowledge and a deeper understanding of human biology and genetics. The wide repertoire of structural information now available from reliable and accurate prediction methods provides the opportunity to investigate the molecular origins of most of the rare diseases reviewed in the Orpha.net database. Thus, it has been possible to analyze the topology of the pathogenic missense variants found in the 2515 proteins involved in Mendelian rare diseases (MRDs), which form the database for our structural bioinformatics study. The amino acid substitutions responsible for MRDs showed different mutation site distributions at different three-dimensional protein depths. We then highlighted the depth-dependent effects of pathogenic variants for the 20,061 pathogenic variants that are present in our database. The results of this structural bioinformatics investigation are relevant, as they provide additional clues to mitigate the damage caused by MRD.

## 1. Introduction

Rare diseases (RDs) are defined by the World Health Organization as affecting fewer than 65 per 100,000 people, a characteristic that is mainly responsible for the lack of knowledge, expertise, and, therefore, effective treatments. Today, RD is emerging as a public health priority, and an increasing number of international networks are active to increase its visibility at the global level and to expand and share research, medical, and social care strategies. The fact that more than 70% of RDs are of genetic origin [1], and, therefore, the same DNA mutation is present in each cell type, means that a wide variety of effects occur in the affected human body. As a result, Mendelian diseases (MRD) are almost impossible to cure, although there are approaches to treat or manage some of the associated signs and symptoms [2]. If the molecular origins of MRD can be attributed to missense variants, and thus to well-defined changes at the protein level, we could, in principle, explore the correlations between the protein structural changes that occur, and the abnormal functions observed to develop rational therapeutic strategies. It is interesting to note that missense pathogenic variants are very common, as they occur in about half of the items that are present in the ClinVar genomic variant database [3]. The assignment of protein mutation sites for genomic missense variants to surface, core, or interaction regions has been proposed [4] when structural information is available from the Protein Data Bank [5]. However, despite the large number of known protein sequences, the limited number of experimentally resolved protein structures is a significant barrier to studying the structure–function correlation of proteins involved in MRD. Artificial intelligence (AI) has recently partially overcome the problem of limited structural information for investigating the effects of molecular changes on protein function [6]. Millions of reliably calculated protein structure models are currently available in the freely accessible AlphaFold database (https://alphafold.ebi.ac.uk/, (accessed on 14 October 2023)), providing broad coverage of the entire content of UniProtKB, the standard repository for protein sequences and annotations [7]. In addition, AI has recently developed AlphaMissense, a new powerful tool for predicting pathogenicity scores for all observed missense genomic variants [8]. Thus, structural bioinformatics can operate efficiently to provide powerful shortcuts for suggesting the protein basis of pathologies at the atomic level and possible remedies for MRD, as we describe in the present report with the implementation of a procedure to scan the database provided by Orpha.net, which correlates each MRD point with the corresponding mutated gene [9]. Orpha.net is therefore a suitable starting point for a structural investigation routine, which we have named Orphanetta (Orpha.net topological analysis). Orphanetta provides a general network of molecule-based information about the structural features of MRD pathogenic variants. It can therefore be a powerful tool to guide the search for new potential treatments of any pathology, present in the Orpha.net database, having structurally defined missense mutation sites. 

## 2. Results

**Figure 1:**
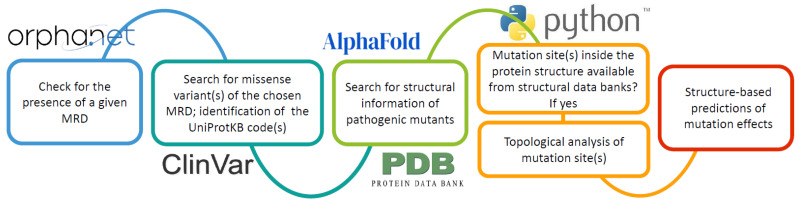
The Orphanetta workflow.

The Orphanetta workflow is summarized in Figure 1.

As of 10 October 2023, the Orpha.net database listed 4338 genes associated with MRD, which were the starting point for our investigation. ClinVar incorporated all the latter genes, adding the relevant information on the molecular consequence of their reported variants. Thus, we have delineated 3145 missense-mutated proteins involved in MRD that represent the target of our structural analysis.

### 2.1. Deriving Structural Information from the Orpha.net Database

**Figure 2:**
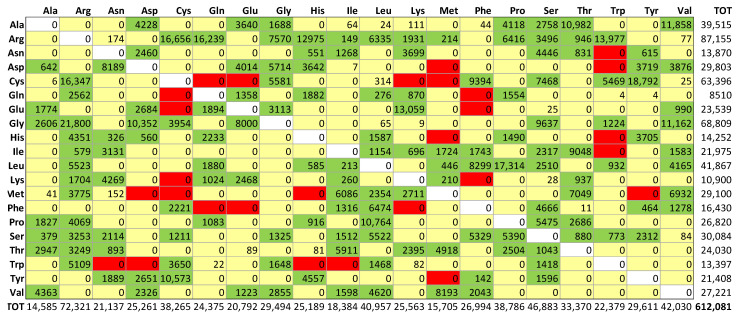
Amino acid distributions of missense pathogenic variants reviewed by Opha.net dataset. Rows describe how each of the natural amino acids has been replaced by column residues. Colors refer to the number of codon nucleotides involved in mutations: green, yellow, and red indicate, respectively, one-, two-, and three-nucleotide changes.

Among all the 3145 proteins that were involved in MRD, several imply impossible double or triple nucleotide codon changes, as we observed in their amino acid replacement matrix, see Figure 2. Then, as we have previously performed in the general case of all the ClinVar missense variants [10], we performed a preliminary removal of the latter anomalous items. Thus, for our structural Bioinformatics analysis, we have obtained a final dataset containing 2797 proteins undergoing MRD pathogenic variants.

Based on the corresponding UniProtKB accession codes, we searched for the presence of each of these 2797 proteins in the Protein Data Bank [5]. As this search yielded only 1738 non-redundant experimentally resolved protein structures, we investigated for the presence of the additional structural information in the database of AlphaFold predicted structures [6]. We have considered only those files that ensured reliable AlphaFold models, i.e., the ones having pLDDT scores higher than 0.8. Accordingly, we have analyzed the structural features of 2515 missense-mutated proteins that form the complete repertoire of our structural Bioinformatics investigation. Multiple MRD pathogenic variants are associated with the latter proteins and the complete list of the structurally defined 20,061 pathogenic variants is given in Table S1.

### 2.2. Topological Assignments of Protein Mutants Responsible for MRD

**Figure 3:**
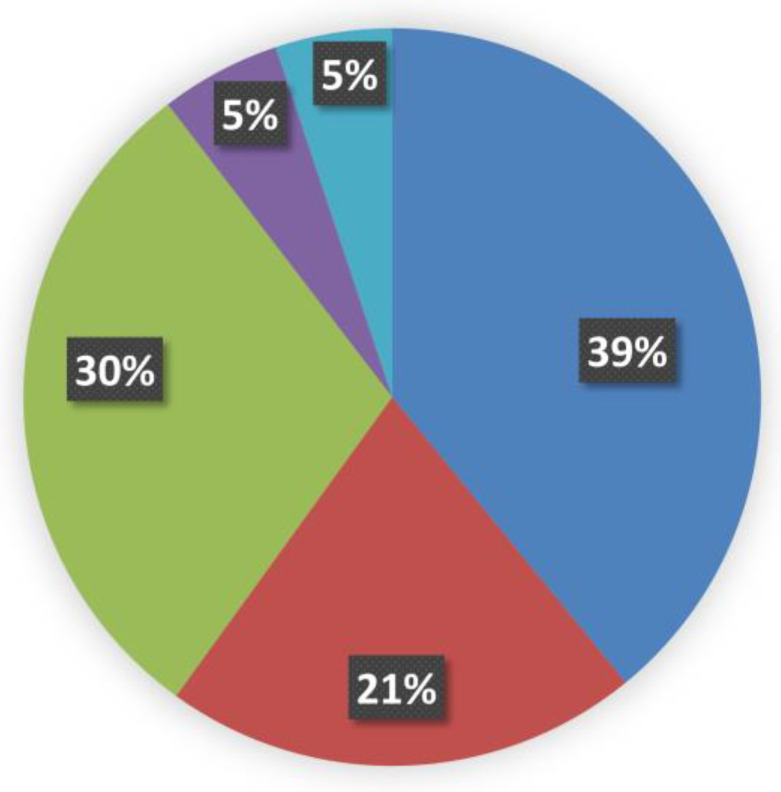
The sterical distribution of mutation sites in MRD proteins of our dataset. Structural data are clustered according to the POPS algorithm [4], and the Q(SASA) parameter categorizes the 20.248 mutation sites as follows: (i) 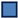 fully buried < 0.15; (ii) 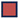 internal > 0.15 and <0.24; (iii) 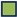 intermediate > 0.24 and <0.60; (iv) 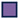 external > 0.60 and <0.80; and (v) 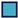 fully solvent-exposed > 0.8.

The effect of amino acid replacements on protein evolution has been considered since the times when protein structural information was limited to a handful of experimentally resolved examples [11,12,13]. Nowadays, the wealth of the available protein structures, both experimentally obtained and high-quality predicted, allows us to discuss amino acid replacements also in terms of their 3D location. Thus, the structure-based analysis can yield powerful information for understanding the molecular mechanisms of diseases [14]; we carried out the topological analysis of mutation sites that are present in each of the 2515 proteins of our dataset to distinguish the outer and inner locations of the amino acid replacements by using POPScomp [15]. Hence, the Q(SASA) parameter has been used to assign the mutation site topology from protein cores to their most external regions [4]. Accordingly, Q(SASA) values lower than 0.15 have been considered diagnostic of inner positions of the missense mutation site, and the topology of all the variants related to MRD diseases are distributed as reported in Figure 3 and Table S1.

**Figure 4:**
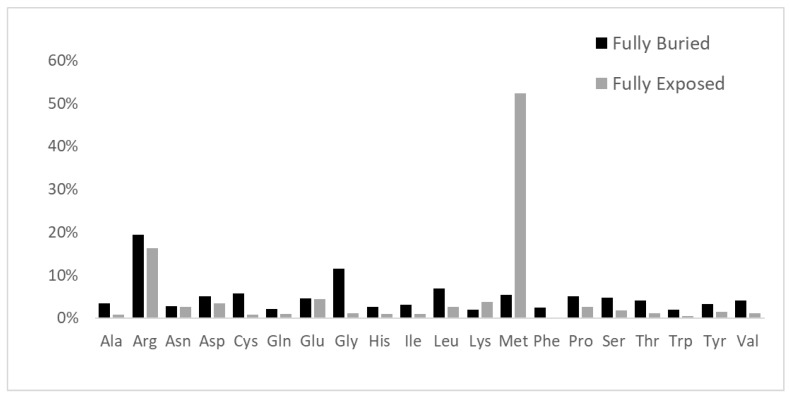
Profile of amino acid replacements in the structurally defined proteins that are responsible for MRD. Histograms refer to percent of amino acid variations in the fully buried and fully exposed regions, respectively, in black and grey colors.

All the internal mutation sites related to MRD represent the very large majority of all the variants, in agreement with previous suggestions for the incidence of inner residues in general on pathogenicity [16]. Data reported in Figure 4 clearly show how the replacement profiles of amino acids are quite different in the case of buried or exposed mutation sites.

**Figure 5:**
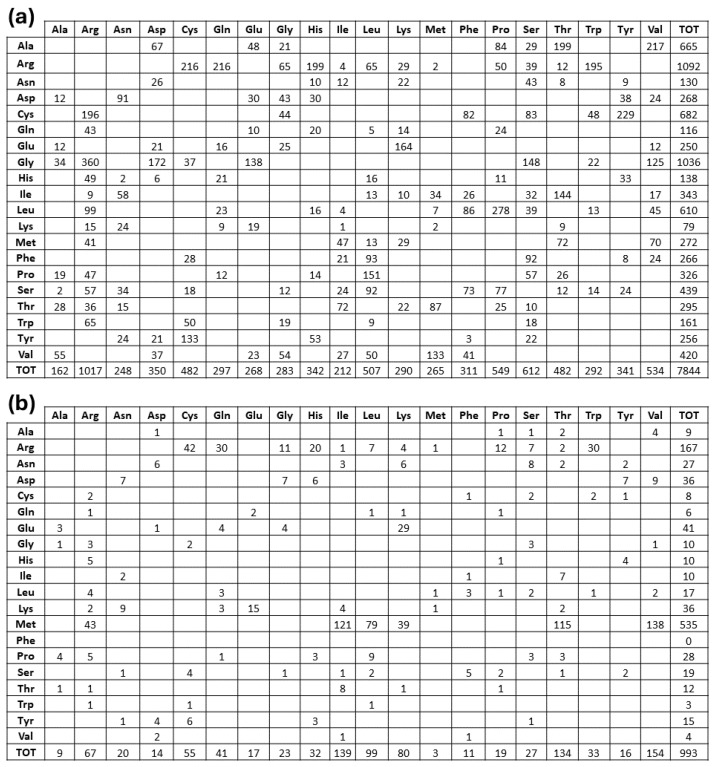
Amino acid replacement matrices of fully buried (**a**) and fully exposed (**b**) pathogenic variants. As in Figure 3, rows describe how each of the natural amino acids has been replaced by column residues.

Furthermore, from inspection of the amino acid replacement matrices of the latter two groups of variants, shown in Figure 5, several features are worth a preliminary discussion. As far as fully buried mutation sites are concerned, Gly and Arg are equally abundant and much more frequent than all the other replaced amino acids, see Figure 5b, and Cys, despite its low occurrence in proteins, exhibits a very frequent involvement in pathogenicity. In the case of fully exposed mutation sites, see Figure 3 and Figure 4, a main signal arises from the observation that very frequent substitutions occur for Met and Arg, representing, respectively, 53% (a total of 535 in the fully exposed regions) and 17% (a total of 1092 in the fully buried regions) of the total ones. The relevance of these findings in relation to MRD onset is underlined in Section 3 of the present report.

### 2.3. Predicting the Structural Effects of Specific Amino Acid Replacements 

The mutation matrices shown in Figure 5 confirm that MRD pathogenicity arising from amino acid substitutions in the proteins of our dataset is not univocal, but it is determined by the topology of the mutation site. For instance, replacements of amino acids bearing hydrophobic bulky side chains with other ones having electric charges, in the case they occur in the protein interior, determine a disruption of the folding nucleus, and the unfolded protein undergoes a fast proteolytic digestion. Whenever the same type of event occurs in the solvent-exposed protein surface, the protein folding process is fully conserved, but this feature causes a strong change in the protein interaction pattern with its molecular environment, ranging from the inhibition of protein quaternary assemblies to changes in protein–ligand interactions. In general, the effects of an amino acid substitution can determine the (i) reduction in the structural stability, (ii) inhibition of folding nucleus formation, (iii) interference in protein–protein, protein–nucleic acids, or protein–ligand interactions. To predict the effect of amino acid replacements, they are usually grouped into four main categories, which must undergo further subdivision to account for their hydrophobicity, polarity, electric charge, and side chain size. Hence, we have considered 10 different subgroups of amino acids, as reported in Table 1. 

Thus, possible effects of the amino acid variations in the structurally characterized proteins of our Orpha. net-derived datasets are predicted and listed in Table S1. In Table S2 are listed all the 5221 MRD reviewed from Orpha.net, whose structural origins can be tracked with the Orphanetta procedure.

## 3. Discussion

The genetic information offered by the Orpha.net database has been our starting point for collecting additional clues at a molecular level for possible MRD remediation. Our topological analysis of all the amino acid replacements that are correlated to MRD, clearly confirms what has been already observed in general [17], i.e., low solvent exposure of mutation sites is mainly correlated to the onset of genetic diseases.

Data shown in Figure 5 indicate the very high frequency of pathogenic variants found for Arg and Gly in protein cores. The presence of Arg in inner protein regions, indeed, is very critical to maintain a positive charge, when it is needed in hydrophobic environments or to bind internal water molecules [18]. Thus, MRDs frequently come from pathogenic variants of Arg, a residue that has CG (U, C, A, G) codons that are particularly unstable [16,19]. It is well known that Gly residues are largely conserved, as with their small dimensions they can play unique roles in the structure of folded proteins [20,21]. As shown in Figure 5a, the core glycines of our structural dataset are mostly replaced by charged amino acids or residues with larger side chains, in both cases perturbing the folding nucleus formation. Figure 5a also evidences how frequently Cys substitutions in the inner protein region are lethal for the folding process, as they interrupt the cysteine-bridge network, which is necessary to stabilize the correct protein structure. Upon defining fully exposed protein residues exhibiting a Q(SASA) value above 0.8, we have selected 1.002 mutation sites. They are mainly due to Met and Arg substitutions, respectively, at 53 and 17%. It is worth noting that pathogenicity always occurs whenever the mutation involves a surface-exposed Met occupying the amino terminus position. This finding is in total agreement with a recent investigation [22] that underlines how such a mutation in the signal peptide interferes with protein targeting, translocation, processing, and stability. Several very pathogenic effects can arise from replacements of Arg occupying protein surface positions, as the latter amino acid drives most of the protein interactions with other proteins, nucleic acids, and ligands. As reported in Figure 5b, there are 167 cases where Arg is changed into all the possible alternatives given by single nucleotide variations. It would be precious information to know when these Arg mutation sites occur at the interface with their molecular partners for a better understanding of the mechanisms of pathogenicity. Very likely, this task will be possible in the near future thanks to artificial intelligence procedures such as AlphaFold-Multimer [23]. Thus, from any of the MRDs listed in Table S2, we can find the structurally defined protein(s) involved in missense pathogenic variants. Then, from the UniProtKB code(s) reported in Table S1, the mutation site topology can be delineated to predict the structure and function damage caused by the amino acid replacement. 

## 4. Materials and Methods

### 4.1. Dataset of Missense Variants

As of 10 October 2023, the Orpha.net database (https://www.orpha.net/, (accessed on 10 October 2023)), providing a standardized classification and coding system for all the known rare diseases, listed 4338 genes associated with MRDs. This information represents the starting point for the present investigation. ClinVar databank [3] incorporated all the latter genes, adding the relevant information on the molecular consequence of the reported variants. Thus, we have delineated 3145 pathogenic missense variants involved in MRD that represent the target of our structural analysis. 

### 4.2. Structural Analysis

To complete the dataset of the present structural analysis, the Protein Data Bank (PDB) [5], a widely used repository for 3D structural data of biological biopolymers, was accessed to retrieve structural information for MRD pathogenic variants. In addition, AlphaFold [6] was used to obtain structural information on the proteins that were not present in the PDB. AlphaFold’s algorithm analyzes the amino acid sequence of a protein to predict the distances between pairs of amino acids, which are then used to generate a 3D model of the protein structure. For each residue, AlphaFold outputs a predicted Local Distance Difference Test (pLDDT) score, to assess the reliability of specific regions within the structure of interest. In the present investigation, we have considered only predicted models possessing very high reliability with a pLDDT > 0.8. The effects of amino acid replacements are only briefly discussed in terms of the corresponding site topology. Specific analysis of the structural changes due to mutations would require molecular dynamics simulations and could be performed only for single MRD cases. The latter methodology, indeed, needing large computational resources and times, is not suited for high-throughput investigations. 

### 4.3. Atom Depth Calculations

The POPScomp program [15] computes the Solvent Accessible Surface Area (SASA) of any structure in a suitable PDB format. Q(SASA), which represents the ratio between the SASA of a generic Xyz amino acid inserted in the protein structure and a reference value related to the SASA of the amino acid side chain in a tripeptide GlyXyzGly. Based on the Q(SASA) parameter, it has been possible to assign the mutation site topology: (i) fully buried < 0.15; (ii) internal > 0.15 and <0.24; (iii) intermediate > 0.24 and <0.60; (iv) external > 0.60 and <0.80; and (v) fully solvent-exposed > 0.8.

## 5. Conclusions

The Orphanetta procedure, summarized in Figure 1, by linking the genomic information provided by the Orpha.net database to the ones available from structural data banks, seems to be well suited for AI developments that can yield fast and automatic answers for deciding the priorities for genomic editing to solve or at least mitigate the effects of MRD.

Despite this potential, this study acknowledges limitations associated with public databases. ClinVar database may be limited by inconsistencies in variant interpretation and the varying quality of submissions from different sources, which have been, however, addressed over time [24]. The Orpha.net database, instead, relies on voluntary reporting from various sources, which can lead to potential gaps or inaccuracies in the information on rare diseases, affecting the reliability and comprehensiveness of this study’s findings. Future research on MRDs must undoubtedly continue, including the interpretation of findings regarding the implications of variants on protein function and their contribution to MRD pathogenicity. As an example, the observation of replacements of amino acids bearing bulky side chains with glycine in protein outer regions yields pathogenic modifications of protein surface dynamics that could be restored simply using suitable ligands [10]. These efforts will be crucial in developing targeted therapies that can mitigate the effects of these molecular disruptions, ultimately improving patient outcomes.

## Figures and Tables

**Table 1:** Physico–chemical properties of natural amino acids. Sizes of amino acid side chains are classified according to ref. [17].

Electrically Charged Side Chains	Polar Uncharged Side Chains	Hydrophobic Side Chains	Special Cases
Positive: Arg, His, Lys Negative: Asp, Glu	Small size: Ser, Thr Large size: Asn, Gln, Tyr	Small size: Ala, Val Medium size: Ile, Leu, Met Large size: Phe, Trp	Cys; Gly; Pro

## Data Availability

The data presented in this study are available on request from the corresponding author.

## Supplementary Materials

The supporting information can be downloaded at: https://www.mdpi.com/article/10.3390/ijms25136953/s1.
