# Supplementary material for: Molecular Origins of the Mendelian Rare Diseases Reviewed by Orpha.net: A Structural Bioinformatics Investigation
Source: Int J Mol Sci. 2024 Jun 25;25(13):6953. doi: 10.3390/ijms25136953 (PMC11241713; doi:10.3390/ijms25136953)
Supplement: Supplementary file 1 [file ijms-25-06953-s001.zip › orphanetta-SI.pdf]

Supporting Information for

Molecular Origins of the Mendelian Rare Diseases Reviewed by Orpha.net: A Structural Bioinformatics Investigation

Table S1. Topology of mutation sites of UniProtKB coded proteins involved in MRD. Preliminary notes on the possible effects of amino acid replacements on protein structures and dynamics are given.

Legend:

Associated effect	N°
Change in protein-ligand interaction network	1
Change in protein-ligand interaction network or aggregation	2
The folding nucleus cannot be formed	3
Loss of structural stability	4
Major structural change	5
Minor structural change	6
Possible protein aggregation	7
No detectable effect	8
Minor influence on protein core	9
Possible aggregation	10
Protein aggregation	11
Protein core perturbation	12
Electric charge change	13

See the attached Excel file “Table S1.xlsx”

Table S2. List of MRD related to missense pathogenic variants of *Orphanetta* defined proteins.

See the attached Excel file “Table S2.xlsx”
